# Operationalising sexual and reproductive health and rights in sub-Saharan Africa: constraints, dilemmas and strategies

**DOI:** 10.1186/1472-698X-11-S3-S8

**Published:** 2011-12-16

**Authors:** Rose Ndakala Oronje, Joanna Crichton, Sally Theobald, Nana Oye Lithur, Latifat Ibisomi

**Affiliations:** 1 Institute of Development Studies, University of Sussex, Brighton, BN1 9RE, UK; 2 African Institute for Development Policy (AFIDEP), Royal Offices, Mogotio Road off Chiromo Lane, Westlands, Nairobi, Kenya; 3 School of Social and Community Medicine, University of Bristol, Canynge Hall, 39 Whatley Road, Bristol, BS8 2PS, UK; 4 Liverpool School of Tropical Medicine, Pembroke Place, Liverpool, L3 5QA, UK; 5 INDEPTH Network, 11 Mensah Wood Street, East Legon, Accra, Ghana; 6 Demography and Population Studies Programme, University of the Witwatersrand, Private Bag 3, WITS 2050, Johannesburg, South Africa

## Abstract

**Background:**

The continued poor sexual and reproductive health (SRH) outcomes in sub-Saharan Africa highlight the difficulties in reforming policies and laws, and implementing effective programmes. This paper uses one international and two national case studies to reflect on the challenges, dilemmas and strategies used in operationalising sexual and reproductive health and rights (SRHR) in different African contexts.

**Methods:**

The international case study focuses on the progress made by African countries in implementing the African Union’s Maputo Plan of Action (for the Operationalisation of the Continental Policy Framework for Sexual and Reproductive Health and Rights) and the experiences of state and non-state stakeholders in this process. The case was developed from an evaluation report of the progress made by nine African countries in implementing the Plan of Action, qualitative interviews exploring stakeholders’ experiences and perceptions of the operationalisation of the plan (carried out as part of the evaluation) in Botswana and Nigeria, and authors’ reflections. The first national case study explores the processes involved in influencing Ghana’s Domestic Violence Act passed in 2007; developed from a review of scientific papers and organisational publications on the processes involved in influencing the Act, qualitative interview data and authors’ reflections. The second national case study examines the experiences with introducing the 2006 Sexual Offences Act in Kenya, and it is developed from organisational publications on the processes of enacting the Act and a review of media reports on the debates and passing of the Act.

**Results:**

Based on the three cases, we argue that prohibitive laws and governments’ reluctance to institute and implement comprehensive rights approaches to SRH, lack of political leadership and commitment to funding SRHR policies and programmes, and dominant negative cultural framing of women’s issues present the major obstacles to operationalising SRH rights. Analysis of successes points to the strategies for tackling these challenges, which include forming and working through strategic coalitions, employing strategic framing of SRHR issues to counter opposition and gain support, collaborating with government, and employing strategic opportunism.

**Conclusion:**

The strategies identified show future pathways through which challenges to the realisation of SRHR in Africa can be tackled.

## Introduction

More than 15 years after the radical shift in policy from a focus on population control to a focus on individual needs and rights initiated at the 1994 International Conference on Population and Development (ICPD), sexual and reproductive health and rights (SRHR) indicators remain poor in sub-Saharan Africa (SSA). Specifically, the ICPD made advancing gender equality, eliminating violence against women, ensuring women’s ability to control their own fertility, and universal access to sexual and reproductive health (SRH) information and services cornerstones of population and development policies [1]. At the conference, 179 countries agreed to implement the ICPD Programme of Action.

Although some progress has been made in SSA in terms of developing reproductive health policies and reforming laws to provide a framework for the implementation of SRHR programmes, SRHR still remain non-priority issues on the development agenda of many SSA countries due to limited political leadership and commitment to the realization of SRHR, and inadequate resource allocation [2-4].

Consequently, in much of SSA, maternal mortality and morbidity remain unacceptably high, unsafe abortion claims an estimated 22,000 lives of women each year, contraceptive prevalence is low (varying between 10-50% among women in union), early marriages and teenage pregnancy persist, and gender inequities and incidences of gender-based violence remain high [5].

It’s important to note that the difference between SRH and SRHR often leads to confusion at both policy and programme levels thereby presenting a barrier to operationalisation. Further, the lack of a universally recognised definition of SRHR at the international level [6] is another challenge for implementing national policies and programmes to realise these rights. As used in this paper, SRH refers to everything encompassed in both sexual health and reproductive health, as defined by the ICPD 1994. The ICPD included sexual health as part of reproductive health and defined reproductive health as ‘a state of complete physical, mental, and social well-being and not merely the absence of disease or infirmity, in all matters relating to the reproductive system and to its functions and processes. Reproductive health therefore implies that people are able to have a satisfying and safe sex life and that they have the capacity to reproduce and the freedom to decide if, when and how often to do so’ [1]. SRHR, on the other hand, is based on international human rights law and is about the right to SRH information, services and autonomy. However, SRH-related human rights are spread throughout various international Human Rights frameworks and are interpreted in a range of ways by different stakeholders. The lack of a universally recognised definition of SRHR at the international level [6] presents a challenge because SRHR covers a range of rights of varying levels of controversy, which can lead to confusion. SRHR, as used in this paper, is understood as the right for all, whether young or old, women, men or transgender, straight, gay, lesbian or bisexual, HIV positive or negative, to make choices regarding their own sexuality and reproduction, providing these respect the rights of others to bodily integrity [6]. This definition also includes the right to access information and services needed to support these choices and optimise health [6].

As mentioned above, some SRHR issues – provision of safe abortion, provision of SRH information and services to adolescents, sexual orientation and identities (Lesbian, Gay, Bisexual, Trans-gender, and Intersex), access to SRH services by people living with HIV/AIDS (PLWHAs), and sexual violence against women and girls – remain controversial in most countries. The controversies arise from their contradiction with certain cultural, religious and individual beliefs, norms and values. Thus, efforts to change SRHR policy often receive strong opposition from certain political, religious and community leaders. Given this ‘hostile’ environment, many African governments either shy away from addressing these issues or take discriminatory approaches in policy-making and legislation [7].

As the contentiousness of certain SRHR issues persists compounded by the confusion between SRH and SRHR, these conditions continue to cause human rights violations, illness and even deaths, in addition to affecting other development indicators [2]. Despite these policy constraints and setbacks, a number of stakeholders (including some government officials, human rights groups, women’s rights movements, donors, and researchers) continue to push for getting contentious SRHR issues on the government agenda in different countries.

Using one international and two national case studies, this review paper reflects on the constraints, dilemmas and strategies used for getting controversial SRHR onto the policy agenda and influencing decision-making in different African contexts. The authors pool learning from these three case studies to highlight the strategies that different stakeholders can use to work their way around the opposition to contested and complex SRHR issues in different policy arenas.

The purpose of this paper is not just to share experiences and lessons, but also to contribute to the debate on challenges and opportunities for bringing controversial SRHR issues onto the agendas of government in SSA and influencing decision-making on these issues. Literature on policy processes has shown that policy change is not simply a technocratic process based on rational analysis, but a profoundly political process that is complex, messy and power-laden [8]. It has been argued that issues get onto the government’s agenda when three streams intersect – *problems*, *policy* and *politics *[9]. The intersection can happen by chance and/or through the activities of different policy actors [9]. Some studies have revealed the important role government policy actors can play in bringing about policy change [10], while others have emphasized the role of policy coalitions in policy change [11]. Still, others have argued for the important role of ideas, framing, and use of policy narratives in bringing about policy change [12,13]. The discussion of the case studies will explore their linkages with the international literature on agenda setting and policy change.

## Methods

This paper adopts a case study approach combined with a review of literature. The case study approach is used because not much is known about the ways that different stakeholders negotiate the challenges and dilemmas in operationalising SRHR in SSA, and the case study approach has been noted as being particularly appropriate for researching an area where few studies have been carried out [14]. The international case study focuses on the progress made by African countries in implementing the African Union’s Maputo Plan of Action and the experiences of state and non-state stakeholders in this process. The case is developed from an evaluation report of the progress made by nine African countries (Botswana, Burkina Faso, Cameroon, Ghana, Ethiopia, Nigeria, Rwanda, Senegal, and Uganda) in implementing the Maputo Plan of Action (including the constitutional and policy environment), and analysis of qualitative interview data from the evaluation exploring stakeholders’ experiences and perceptions of the operationalisation of the plan in two countries (Botswana and Nigeria). The interviews in the two countries were purposively conducted to include stakeholders operating at different levels and with different interests and spheres of influence in relation to the operationalisation of the plan. Participants were identified at a regional level (e.g. African Union) , sub-regionally (e.g. SADC) and nationally, including both national government officials whose work focuses on the broad arena of SRHR (for example representatives from Ministries of Health, Finance and Women’s Affairs or Gender, donors and civil society organisations involved in SRHR-related activities). The first national case study explores the processes involved in influencing Ghana’s Domestic Violence Act passed in 2007 by the Ghanaian parliament. This case is developed from a review of scientific papers and organisational publications that detail the processes involved in influencing the Act, and a qualitative interview with one of the experts involved in influencing the Act. The second national case study examines the experiences with introducing the 2006 Sexual Offences Act in Kenya, and it is developed from organisational publications on the processes of enacting the Act and a review of media reports on the debates and passing of the Act. The authors draw on their experiences and reflections to complement the data from other sources since all five authors have been involved either in conducting the studies reviewed in developing the case studies or have been extensively involved in SRHR research and influencing SRHR policy processes in African countries. The authors also draw on media reports on contentious SRHR issues in SSA.

Why were these three cases selected? The international case study was selected because it examines the legal and policy environment as well as programme implementation for SRHR across SSA. The Ghana and Kenya cases feed into the international case study by providing examples of the legal and policy constraints and the legislating processes that bring about change on key SRHR issues.

## Findings: case studies

### The evaluation of African Union’s sexual and reproductive health framework, the Maputo Plan of Action

The Maputo Plan of Action was launched in 2006 to implement the Continental Policy Framework on SRHR adopted by the Conference of African Ministers of Health in October 2005 in Gaborone, Botswana. It calls on African Union (AU) member states to enact policies, advocate for SRHR, build the capacity of health care providers and expand access to reproductive health services in partnership with civil society organisations, the private sector and development partners [15]. The Plan covers the period 2007-2010. In 2008-9, an evaluation of the implementation of the Maputo Plan of Action conducted by the African Population and Health Research Centre [4] found that although most countries have formulated SRH policies, they have not necessarily translated these into the provision of services. It was also found that CSOs are demonstrating versatility and strong leadership in their SRHR work, which contributes towards the achievement of the goals of the national SRH policies and the Maputo Plan of Action. Further, it found that the language of rights in SRH is still controversial in African countries and continues to undermine SRHR policy and programmes. In addition to these findings, the case study examines the experiences of actors in Botswana and Nigeria drawn from the larger qualitative data set gathered by the evaluation study combined with media reports on contentious SRHR issues in SSA. These experiences are summarised here below.

#### The human rights approach to SRH still poses challenges in operationalisation

African countries have put in place reproductive health policies and even though some level of the human rights language has been incorporated into the policy documents, implementation remains problematic [4]. Our analysis of interview data showed that in Botswana and Nigeria, there are still challenges with access to SRH information and services, especially by minority groups such as People Living With HIV and AIDS (PLWHAs), adolescents, prisoners and LGBTs. Stakeholders in Botswana and Nigeria felt that the rights approach to SRH was not supported by their governments. For example:

‘Government is reluctant to support human rights. It closes down when it comes to issues of rights’. (In-depth interview respondent, Botswana CSO).

This lack of support however is often due to the government’s fear of backlash from the public. As a representative of a foundation in Nigeria noted, one of its main challenges was:

‘Getting State Governors to co-invest in SRH initiatives … Politicians fear backlash if they publicly support SRH’. (In-depth interview respondent, donor foundation, Nigeria).

Botswana respondents noted that there was little support for the SRH rights of people living with HIV/AIDS in the country. They observed that HIV positive (HIV+) women who became pregnant were often accused of spreading the virus and were shunned by healthcare workers, yet they too had a right to receive healthcare. A community-based organisation (CSO), which took up this issue, found that healthcare workers were not equipped to talk about SRH with PLWHA apart from recommending condom use, and it was implied that they discouraged HIV+ women from getting pregnant.

Respondents from the Botswana Ministry of Health (MoH)’s SRH department indicated that although there were clinics in prisons to provide SRH services, such clinics did not provide condoms since prisoners ‘*are not expected to be having sex’*. These issues are aptly captured by a respondent from the Botswana CSO, who observed that the country did not have:

‘a legal framework/policy for PLWHA, prisoners and homosexuals on how to access SRH services. Service providers are not allowed to supply commodities to prisoners and homosexuals are not recognized by the government.’ (In-depth interview respondent, Botswana CSO).

In most of sub-Saharan Africa, homosexuality is prohibited by law and is a punishable crime. Homosexuality attracts condemnation from church leaders and sections of the public based on the argument that the practice is immoral and ‘un-African’. Recent media reports on homosexuality around Africa have revealed both government and public opposition to the recognition of, and provision of services to, people in same sex relationships. Media coverage revealed that members of the public in Kenya prevented a would-be first gay wedding from occurring when they stormed the house where the gay couple lived and forced the couple out moments before the wedding [16]. In October 2010, a call by Kenya’s Minister for Gender to the public to accept gays was strongly condemned by religious leaders who described the Minister’s call as ‘satanic and contrary to African culture’ [17]. In Malawi, a gay couple was jailed for holding a wedding and only pardoned following an uproar from the international human rights community [18]. Uganda is in the process of legislating against pornography in the mass media, and sections of the proposed law seek to outlaw homosexuality [19]. This conservative environment on the continent continues to hinder LGBTs’ access to SRH information and services.

#### Adolescents’ access to sexual and reproductive health information and services is often opposed

On operationalising adolescent SRHR, a Nigerian CSO noted that its programme - the Family Life HIV Education (an SRH curriculum being implemented in schools in some Nigerian states) - was initially opposed by parents and it had to be reframed and some components removed to gain support. The CSO respondent observed that:

‘[T]here was uproar against it when we first conceived it as “National Sexuality Education Curriculum,” and in response we removed certain sexuality issues such as masturbation and also changed the name to “Family Life HIV Education,” with States being guided to implement it as per their cultural peculiarities and contexts.’ (In-depth interview respondent, Nigerian CSO).

Similarly, a respondent from the Botswana National Youth Council noted that earlier programmes initiated by its members on adolescent SRHR were opposed by parents. To get parents’ support, organisations had to contextualise the programmes in relation to HIV/AIDS and rape. They also had to establish youth-adult partnership projects, as well as avoid focusing only on young people, especially given that adults were often barriers to young people’s access to SRH information and services.

Respondents from an international development agency in Botswana noted that although Botswana had a policy on education and access to SRH for young people, the health system was still unfriendly and inaccessible as young people often felt judged when they sought SRH services.

#### Accessing safe abortion remains unachievable

Abortion remains illegal in most African countries and efforts to repeal the law in order to reverse this situation attract strong opposition and controversy. This is despite the fact that 750 out of every 100,000 unsafe abortions in SSA result in death [20]. The opposition to the provision of abortion is grounded in cultural, religious and individual values. Culturally, it is argued that abortion is a ‘foreign’ practice (despite public health evidence to the contrary) and that in Africa, children belong to the society and women should not be given the autonomy in making abortion decisions. Dr. Jean Kagia, a Kenyan gynaecologist and pro-life activist has argued that ‘*there is no community that throws away a child. Even when the mother dies*, *there is always somebody else who will take over that child’ *[21]. A CSO representative in Nigeria indicated that their advocacy for the provision of safe abortion had been ‘ridiculed by parliament’ and the only success they registered was the introduction of ‘safe termination of pregnancy’ in the curricula of colleges of medicine in the country.

Even in countries where abortion laws are less restrictive, access to abortion services is still a challenge because of the negative attitudes of healthcare providers. A representative from the Botswana government’s SRH department noted that although the country’s law allowed access to abortion where the health of the mother or baby was in danger and in cases of rape and incest, its implementation was limited. This observation was supported by a CSO representative who noted that there was a disconnect between the abortion law and practice in Botswana as young people who ‘qualified’ for abortion were often denied services.

These examples depict some of the difficulties in operationalising SRHR in many African countries, and the issue of rights is clearly still to be embraced by many governments. A donor representative in Nigeria summed it up as follows:

‘Nigerian people need to get human rights on the [government’s] health agenda, get politically active and hold their leaders accountable’. (In-depth interview respondent, donor Foundation, Nigeria).

### Influencing Ghana’s domestic violence bill

This case study explores the processes involved in influencing Ghana’s Domestic Violence Act passed in 2007 by the Ghanaian parliament. In 2003, the Ghanaian Attorney General’s office introduced the Domestic Violence Bill in parliament for debate in a bid to legislate against sexual and domestic violence. The bill was greatly opposed and it generated heated debates because it proposed to legislate against the country’s social and cultural systems that tolerated violence against women and girls in the context of gender relations and in the domestic sphere [22]. The bill’s provision on marital rape was especially contentious and its opponents argued that if passed the bill would endanger marriages [22]. The controversy was reinforced by the provisions of section 42(g) of Ghana’s Criminal Code, 1960 (Act 29), which accepts the use of force in marriage on the basis of the consent given upon marriage [22].

To address the controversy, a National Coalition on Domestic Violence Legislation was formed by individuals and human rights organisations which organized from 2003 and carried out a nationwide consultation to win support for passage of the bill [22]. Several strategies were adopted by the Coalition, including a pictorial campaign entitled ‘Faces of Violence,’ comprising a collection of pictures of abused women, projecting ‘voices’ and ‘faces’ of survivors of abuse in the press, a documentary on domestic violence, newspaper articles, radio and TV discussions, and the lobbying of parliamentarians. Parliamentary debates on the bill dragged on for years with several revisions of the bill.

In 2007, the INDEPTH Network Secretariat in Ghana collaborated with one paper author (NOL), a human rights lawyer, to conduct a study in Ghana on the country’s laws and policies governing SRH [23]. The study found that many sexual violence cases could not be prosecuted as victims did not receive the required medical examination due to the prohibitive costs of doing so. The study also established that some health facilities refused to treat survivors who had not been referred to them by the police. Those that were treated did not receive post-exposure prophylaxis from health facilities and were unable to access the few post-traumatic stress services available.

The findings of the study were disseminated widely through the mass media and through meetings with key stakeholders. This coincided with the debates in parliament on the Domestic Violence Bill in 2007, providing an excellent opportunity to influence parliamentarians with the INDEPTH findings and inform the bill so that it could adequately address the issues raised by the research.

The INDEPTH Network therefore set out to engage parliamentarians with the findings of the research. Their dissemination approaches were informed by the understanding that parliamentarians were not easy to access and were also politically driven. Thus, the team assessed parliamentarians’ interests and planned targeted communication around these interests. They identified relevant parliamentarians (those belonging to the Parliamentary Health, Constitutional and Legal, and Gender Committees) and also those that were sympathetic to SRH issues. They worked with parliamentary clerks to identify and organise meetings with parliamentarians. They made short and focused presentations on the issues and what parliamentarians needed to do to address them. The presentations were made by credible and well-known researchers and legal advocates, who also availed themselves for follow-up. Following this dissemination, parliamentarians amended the bill to include a provision that mandates healthcare providers to provide free medical treatment to survivors of sexual abuse and domestic violence, pending a complaint to the police and the issuance of a report. The passing of the bill into an Act in 2007 has provided for sexual violence survivors in Ghana to receive free medical treatment whether or not they have reported a sexual violence case to the police.

The INDEPTH Network noted several important lessons from the influencing process including: clear and concise recommendations directly related to parliamentary mandates could easily be incorporated into bills if well packaged and timed; credible and well-respected message bearers are critical in ensuring message effectiveness; the mass media can be invaluable allies in raising the profile of the issue in focus; policy change does not always lead to practical change – there were complaints that survivors of sexual violence were still charged fees for health care even after the bill was passed, and there is therefore a need to work with the Ghana Health Service to ensure the law on free medical treatment is enforced [23]. Furthermore, Manuh [22] noted that the Act did not explicitly repeal section 42(g) of the Criminal Code 1960 (Act 29) that justifies the use of force in marriage; instead, it provided that ‘The use of violence in the domestic setting is not justified on the basis of consent’. Stakeholders have argued that the retention of section 42(g) of the Criminal Code 1960 (Act 29) ‘reinforces popular notions that marriage serves as an automatic consent to sex, and would make it difficult, if not impossible, for women who experience sexual abuse from their spouses to seek redress’ [22].

Although the INDEPTH Network was not involved in the controversy that dogged the bill since 2003, it played a role in ensuring that the law waived the costs associated with obtaining medical services for survivors of domestic violence.

### The enactment of Kenya’s Sexual Offences Act

This case study examines the experiences with introducing the Sexual Offences Act in Kenya. In 2006, Kenya enacted the Sexual Offences Act to provide a legal framework for dealing with sexual offences and gender-based violence. The Population Council (Kenya office) published a study that documented the processes that led to the enactment of the Act [24]. A draft bill to legislate against sexual violence was first developed by civil society groups and submitted to the Attorney General (AG), who was expected to take ownership of the bill and present it in parliament for discussion. The AG did not agree to present the bill in parliament, but when a parliamentarian (a human rights lawyer) notified the AG’s office that she intended to table a private member’s bill in parliament on sexual offences, the AG formed a taskforce, comprising parliamentarians, civil society groups, and his office, to collate views from different groups and draft the bill [25]. He also provided legal draftspersons to advise the taskforce.

The process of enacting the law witnessed polarised debates in parliament, with many male parliamentarians opposing the bill because they believed it contradicted norms and practices that are central aspects of Kenyan culture. Onyango-Ouma et al. [24] described the parliamentary debate on the bill as being one between ‘traditionalists’ versus ‘liberals’, explaining that ‘the traditionalists did not believe in women’s rights and enjoyment of the same was not permissible’. They noted that most traditionalist parliamentarians represented communities where discrimination against women was culturally entrenched and acceptable, and, as such, these parliamentarians argued that the legislation did not align with their culture.

#### Sexual offences were talked of as acceptable everyday practices

Parliamentarians opposed to the bill described some of the offences in the bill as acceptable everyday practices such as ‘dating’, ‘courtship’ and ‘men’s conjugal rights’. For instance, the offence of unlawful advances was opposed and removed from the bill because the parliamentarians argued that such advances were merely components of sexuality and everyday courtship in the Kenyan society. Marital rape was also opposed and removed from the bill because male parliamentarians argued that there was no such thing as marital rape in African society as both men and women provide consent when making their wedding vows to allow all future sex within the marriage. A male parliamentarian argued that:

*“An activity between a man and his wife in his bedroom cannot …be constituted to be rape. Many people believe …this is not an African issue. Marriage creates sexual license to each party… that is the license they get by saying I do.” *[24]

#### Sexual offences were trivialised

Debates on the sexual offences bill were trivialised by male parliamentarians who justified rape, asserting that when a woman says ‘No’ she means ‘Yes’. Others argued against it, saying that punishing men for sexual offences was the women’s way of ‘*fixing men*’ or ‘*settling scores*’ with men.

The parliament debates resulted in several revisions of the bill and when it was finally passed in parliament, the bill had changed drastically. For example, specific proposals of the bill, including criminalisation of marital rape and sexual harassment, and measures to protect rape victims’ sexual history from being used against them in court, were removed.

Given the enormous opposition to the law, a number of strategies were used to mobilize support from parliamentarians and members of the public, including organising meetings and workshops with opposing groups, media visibility of sexual violence incidents and framing of debates, public demonstrations by civil society, lobbying and advocacy, nationwide campaigns to educate the public on the bill, and forming and working through coalitions [24]. Statistics on the increase of rape cases and stories of rape survivors were used as part of these strategies to make the case for the law. The campaign was carried out by the parliamentarian who led the legislative process in parliament and the civil society organisations involved in the bill. The actors employed different framings of sexual offences to gain support. For instance, they shifted the focus from sexual offences in general to sexual offences against minors and grandmothers. Further framing of the issue focused on the compelling argument that the bill was meant to protect ‘daughters, sons, wives and mothers’, people who were close and dear to everyone, including those opposed to the bill. Among the strategies employed to gain support, public demonstrations were noted to have had negative repercussions by increasing opposition to the bill [26]. However, alliances among parliamentarians, human rights lawyers, scientists and child and women’s rights activists were found to be more effective. Compromises through the removal of certain sections of the proposed bill also increased parliamentarians’ support for the bill.

Key lessons learned from the legislative process included: the need to have broad consultations with diverse actors; coordination and harmonisation of efforts of different actors which resulted in the joint ownership of the process; the need for a good understanding of the legislative process by all stakeholders; the importance of working with the process through government; the important role of an effective negotiator in leading the process; and the importance of packaging the bill or proposal comprehensively knowing that the policy process will involve trade-offs and compromises [24].

## Discussion

The three case studies described above offer experiences for reflection on operationalising SRHR in SSA. Drawing on the case studies, this section discusses the constraints, dilemmas, and strategies used by different groups to try to operationalise SRHR in SSA.

### The constraints to the operationalisation of SRHR in Africa

The three case studies reveal three main constraints to formulating and operationalising SRHR policies and laws in SSA, namely, prohibitive laws and government’s reluctance to institute and implement comprehensive rights-based approaches to SRHR; lack of political leadership and commitment to funding SRH policies and programmes; and the dominant negative cultural discourses on SRHR.

#### Prohibitive laws and governments’ reluctance to embrace SRH rights

Prohibitive laws in most African countries greatly hinder the operationalisation of SRH and rights. For instance, the Maputo Plan of Action evaluation report noted that in most African countries abortion is prohibited except when a mother’s life is in danger [4]. Further, sexual relationships among people of the same sex are often prohibited. Such laws hinder the realization of SRH rights such as access to safe abortion, and access to information and services by people in same-sex relationships. The international case study revealed the prohibitive legal and constitutional environment in which African countries operate, whereas the two national level case studies exemplified the difficulties in repealing the laws as they relate to sexual violence. Kenya and Ghana did not have an effective legislation against gender-based violence until 2006 and 2007, respectively; Kenya adopted one that only legislated against certain sexual offences and left out others such as marital rape and sexual harassment, whereas Ghana adopted one that failed to explicitly repeal section 42(g) of the Criminal Code 1960 (Act 29) that justifies the use of force in marriage.

The case studies also illustrate African governments’ reluctance to embrace the rights approach to SRH despite the fact that many of them have signed up to the Maputo Plan of Action and, in regard to gender violence, many have ratified the Convention on the Elimination of All Forms of Discrimination against Women (CEDAW). The reluctance is as a result of the controversy that surrounds SRHR issues and political climates that do not support SRHR because of ideological opposition from key stakeholders. For instance, although Kenya’s AG played a key role in facilitating the development of the sexual offences bill, he was reluctant to present the bill in parliament for debate when the civil society groups initially submitted a draft to him [25]. Respondents in Botswana and Nigeria in the international case study indicated that their respective governments were often reluctant to discuss or pursue rights issues in SRH.

#### Lack of political leadership and commitment to funding SRHR policies and programmes

The international case study found that although countries had SRHR policies, these did not translate into programmes and service provision partly because of lack of funding for the implementation of policies. The evaluation found that most African governments either did not fund SRH issues or only allocated very limited resources to aspects of SRHR. Such neglect and lack of prioritisation of SRHR issues by politicians and policymakers indicate the lack of political leadership in tackling these issues. This challenge is made worse by the fact that only a handful of donor organisations still focused on funding SRH; while others had shifted their focus to funding disease specific issues such as HIV/AIDS, tuberculosis, and malaria [27]. This challenge has resulted in not only few SRH programmes, but also in inadequate staffing for handling SRHR issues, and existing staff’s lack of skills and capacity to deliver SRHR services. Family planning commodity shortages have also been common occurrences in many African countries because of limited funding and ineffective logistical systems [27]. Given the low priority accorded SRH by donors, the programmes and activities of CSOs have also been constrained.

#### Dominant negative cultural framing of SRHR

Many African societies are patriarchal and so powerful actors such as religious, political and community leaders frame women and SRHR issues in ways that perpetuate male dominance. The way issues are framed is important in operationalising SRHR because it influences the level of priority they receive [28]. The three case studies reveal the negative cultural framing of women’s SRHR issues (e.g. giving men the ‘right’ to discipline their partners), the acceptable cultural norms pertaining to SRHR (e.g. sexual harassment as a way of courting), and the framing of SRHR as ‘unAfrican’, ‘modern’ or as alien to Africans’ way of life. This framing has greatly hindered the operationalisation of SRHR policies and laws as seen in the Ghanaian and Kenyan experiences with legislating against sexual violence.

The challenge of conceiving SRHR issues as acceptable cultural norms is perhaps best captured in Kenya’s experiences. For instance, the parliamentarians argued that rape in marriage was acceptable. Similarly, in a notorious case, a secondary school deputy principal, making a statement after boys from his school raped and killed 19 school girls and injured another 71 in 1993, said that ‘the boys did not mean any harm, they only wanted to rape’ [7].

On the framing of SRHR as alien to Africa, some studies have noted that efforts to reform SRHR policies and laws in Kenya have often been thwarted by this [29]. Thomas [29] found that debates that saw the repealing of the Affiliation Act in Kenya in 1969 (a short-lived law enacted in 1959 and which granted all single women the right to sue the fathers of their children for paternity support), and other later debates opposing the marriage, divorce and inheritance bills, used the duality of the ‘modern’ versus ‘traditional’ as a powerful framing for safeguarding Kenyan men’s privileged legal position and sabotaging efforts to empower women through the law. Thomas [29] argued that ‘modern’ versus ‘traditional’ framings of women’s issues are grounded in particular visions of gender and reproductive relations which emphasise men’s dominance over women and safeguard men’s privileged legal position. These ways of framing SRHR in SSA influence not only the public, but also the attitudes of healthcare providers towards providing certain SRHR services.

### The dilemmas

The contentiousness of controversial SRHR issues presents stakeholders with dilemmas when it comes to operationalisation. From the international case study, respondents in Botswana (from the SRH department, MoH) indicated that there were SRH services provided in prisons, but such services did not provide condoms since same-sex relationships were illegal in the country. But if prisoners were presenting with STIs, it meant they were sexually active. How can they be reached with services to protect themselves? This is the case in many African countries where same-sex relations are prohibited, and therefore no targeted services are provided to people in these relationships, apart from the SRH services provided for everyone else.

The Kenyan case showed just how influential individuals in patriarchal societies can frame sexuality and women in ways that perpetuate and legitimize gender inequality and deny women opportunities to realize their human rights. This issue is compounded by the fact that since women are part of these cultures, many have internalized their rightlessness [30] and often have limited agency. To respond to this challenge, stakeholders struggle with the question: how do we counter negative framings of women and promote more positive ones that can raise the profile of women’s rights? More importantly, stakeholders struggle with how best to involve men in such efforts, because for the efforts to succeed, men have to be involved and appreciate the value of gender equity and equality.

Related to this is the problem of the language of SRH rights with policymakers. Policy-making is arguably male-dominated in most African countries and given the cultural context, the language of SRH rights evokes negativity and often closes doors to influencing policy. In the international case study, respondents from both Botswana and Nigeria indicated that their government was not supportive of the SRH rights based approach.

Another dilemma relates to compromise. In the Kenyan case study, the proponents of the Sexual Offences Act had to make considerable compromises by deleting several sections of the original bill for it to be accepted and passed in parliament. These changes have been argued to have ‘watered down’ the bill considerably [24]. The question here is: How much should stakeholders compromise in their efforts to operationalise SRHR?

### What strategies have different stakeholders used to operationalise SRHR in Africa?

Despite the constraints and the dilemmas, the three case studies also reveal a number of strategies that stakeholders can take advantage of in their efforts to operationalise SRHR in African countries. We have classified the strategies in four broad categories namely, strategic framing of SRHR issues, forging of strategic alliances, working with the government, and strategic opportunism.

#### Strategic framing of SRHR issues

‘Strategic framing’ is defined as ‘a way of selecting, organizing, interpreting and making sense of a complex reality to provide guideposts for knowing, analysing, persuading and acting’ [31]. Strategic framing, which draws on concepts from discourse analysis, has been applied in eliciting support for public issues that are often not a priority for governments or are controversial. In SRHR, strategic framing is often used to get issues onto the government’s agenda as well as to influence decision-making. For instance, to gain support for the gender mainstreaming agenda in various African governments, bureaucrats have strategically framed gender analysis and mainstreaming as important for better and more efficient health systems as opposed to framing it as important for the realisation of equity and rights [32]. The latter framing risked being counterproductive as it was likely to be seen as a threat to the power of decision-makers (who are pre-dominantly male). Also, it has been noted that advocacy efforts that led to the Kenyan government initiating budgetary allocations for family planning framed family planning as critical for ‘development’ as opposed to ‘population control’ [33].

In our case studies, the power of framing is evident in the dominant cultural discourse that has worked against women realizing their SRHR in SSA. But this, as already seen, presents the opportunity for stakeholders to identify and nurture alternative discourses that not only construct women as important and equally deserving human beings, but also highlight the role of men in promoting SRHR in the African society. This suggestion is not new as such alternative framings have been employed by human rights activists for the rights of women, children and sexual minority groups. However, there is scope for stakeholders to make greater use of positive framings of women that are protective of their entitlements that are already present in African communities. For instance, Izugbara and Undie [34] noted that in the Ubang community of Nigeria, women, whether married or unmarried, remain ‘daughters’ to their natal communities, which reflects their natal communities’ role in protecting their rights regardless of their age or (marital) status. In many African cultures, the extended family and community are expected to intervene in cases of gender violence [35]. Crichton [35] found that in a few cases of intimate partner violence in Kenya, relatives-in-law intervened to stop the violence. Although these existing protective cultural and social norms may not adequately prevent rights violations, they could be drawn on in efforts to change attitudes. They could also help to persuade community or religious leaders to speak out on issues such as domestic violence.

As seen in the three case studies, framing was used to get the support of opponents for SRHR policies, legislations and programmes (i.e. support of parents and adults for youth sexuality programmes in Botswana and Nigeria, and support of parliamentarians and public for sexual violence legislation in Ghana and Kenya). This attests to the power of framing, which has been shown to play a key role in influencing policy change [12]. The cases also revealed that reframing SRH issues involves processes of contestation between proponents of different narratives and that different narratives represent certain interests and marginalise others [13]. In our national case studies, three strategies stood out as effective for supporting strategic framing. One is the use of stories of people’s experiences with sexual violence to shape and support the framing and draw attention to sexual offences. The second is the focusing of attention on the rape of young girls and grandmothers as opposed to the rape of ‘generic’ women in Kenya to indirectly challenge some of the arguments of the opponents and appeal for their support. And, the third is the use of the mass media not only in drawing attention to sexual violence but in propagating positive narratives that counter negative ones. The mass media have the power to set agendas and focus public attention on important issues [36].

#### Forging strategic alliances

Networks and coalitions play an important role in bringing about policy change [11]. Kingdon [9] has argued that different policy actors play different roles in bringing about policy change. As seen in both the national case studies, effective networking and drawing on the expertise of different stakeholders can greatly contribute to bringing about policy and programme reform in the area of SRHR. In Ghana, the INDEPTH Network, a research organisation, worked very closely with a renowned human rights lawyer to bring about change in the country’s Domestic Violence Act. Also in Kenya, alliances between parliamentarians, human rights lawyers, scientists and child and women rights activists were instrumental in the development and passing of the Sexual Offences Act. Furthermore, identifying and working with champions such as sympathetic parliamentarians was instrumental in moving forward the sexual offences legislative processes in both countries.

Reaching out to opponents through multiple avenues is also useful in soliciting their support for contentious SRHR issues. In the Kenyan case, Onyango-Ouma et al. [24] noted that the proponents of the bill reached out to parliamentarians opposed to the bill through fellow members of parliament supporting the bill, church leaders, and parliamentarians’ spouses. The successes realised in Ghana and Kenya reveal the constellations of policy actors that make change in SRHR laws possible.

#### Working with the government

Governments are charged with making and implementing policies and legislation. Even where a government shies away from taking public action on controversial issues, it does not necessarily mean individual government actors are not sympathetic to the issue. In many cases, individual policy actors play a key role in facilitating policy change within their political, bureaucratic and economic constraints, as revealed in the work of Grindle and Thomas [10]. In Kenya, the AG was reluctant to present the sexual offences bill in parliament, but when an MP proposed to move a similar bill in parliament as a private members’ bill, the AG provided important support throughout the legislative process [24].

#### Strategic opportunism

Policy processes are not straight forward and there may be sudden points of opportunity to influence decision making [8]. This necessitates identifying and taking advantage of any opportunity that may arise. In Kenya, the parliamentarian who was leading the motion on the sexual offences bill noticed one day that most of the parliamentarians opposed to the bill were not in parliament. She therefore took the opportunity to have the motion and votes cast, and this may be perhaps what saw the bill pass through parliament as, after this stage, parliamentarians could only amend the bill but not reject it [24]. This supports Kingdon’s [9] element of chance in aligning the three streams of *problems*, *policy* and *politics* to elevate an issue onto the policy agenda. In Ghana, INDEPTH took advantage of the parliamentary debates on the domestic violence bill to gain attention for its research findings.

Also, an important strategy that emerges from the three case studies is taking advantage of entry points provided by government policy frameworks. In most African countries, national plans for education and health provide entry points for partnering with the government in improving access to SRHR information and services to marginalised groups such as adolescents.

## Conclusion

The discussions in this paper attest that there are still huge challenges in the operationalisation of SRHR in SSA. Prohibitive laws and governments’ reluctance to institute and implement comprehensive rights approaches to SRH, lack of political leadership and commitment to funding SRHR policies and programmes, and dominant negative cultural framing of women’s issues present the major obstacles to operationalising SRH rights. But these challenges are not insurmountable as revealed by the successes that stakeholders have realised in their efforts to operationalise SRHR. For instance, stakeholders in Ghana and Kenya have countered the strong opposition to legislate against various forms of sexual violence. In Botswana and Nigeria, progress has been made in enabling young people to access SRH information and services. To achieve these successes, stakeholders have formed and worked through strategic alliances and coalitions, employed strategic framing of SRHR issues to counter opposition and gain support, collaborated with individuals within government, and employed strategic opportunism. The use of evidence in the strategies especially highlighting the extent of the issues (e.g. the magnitude of rape incidents) as well as capturing people’s experiences with these issues, has helped galvanise support. These strategies point to the pathways through which SRH policy and programme change can be realised in African countries.

## List of abbreviations used

AG: Attorney General; APHRC: African Population and Health Research Centre; CEDAW: the Convention on the Elimination of All Forms of Discrimination against Women; CSO: Civil Society Organisation; FGM: Female Genital Mutilation; ICPD: International Conference on Population and Development; INDEPTH: International Network for the Demographic Evaluation of Populations and Their Health in Developing Countries; MoH: Ministry of Health; MP: Member of Parliament; PLWHAs: People Living with HIV/AIDS; RH: Reproductive Health; SSA: Sub-Saharan Africa; SRH: Sexual and Reproductive Health; SRHR: Sexual and Reproductive Health and Rights.

## Competing interests

The authors declare that they have no competing interests.

## Authors’ contributions

RNO, JC and ST participated in the conception of the paper. RNO led in drafting the paper. JC, ST, NOL and LI reviewed and contributed to the revising of the manuscript. All authors have seen and approved the final version.

## Authors’ information

RNO has been involved in policy engagement in African countries to promote uptake of SRHR research for the past six years. JC and ST have conducted research on SRHR issues and been involved in policy engagement to promote research uptake in Africa. NOL is a human rights lawyer who was involved in the INDEPTH Network’s study and the research dissemination processes that influenced Ghana’s Domestic Violence Act. LI was involved in carrying out the evaluation of the progress of nine African countries in implementing the Maputo Plan of Action and has also conducted several SRHR studies in Africa.

## References

[B1] UNFPA ICPD ’94: summary of the programme of action 1995

[B2] Glasier A Gülmezoglu AM Schmid GP Moreno CG Van Look PFA Sexual and reproductive health: a matter of life and death Lancet 2006 368 1595 1607 10.1016/S0140-6736(06)69478-6 17084760

[B3] Glasier A Gülmezoglu A Putting sexual and reproductive health on the agenda Lancet 2006 368 1550 1551 10.1016/S0140-6736(06)69485-3 17084739

[B4] Undie C Ibisomi L Muteshi J Fotso JC Zulu E Implementation of the Maputo Plan of Action: Opportunities and Challenges for CSOs Action in Promoting Sexual and Reproductive Health and Rights in sub-Saharan Africa 2010 Nairobi: APRHC

[B5] UNFPA Sub-Saharan Africa Undated http://web.unfpa.org/africa/overview.cfm

[B6] IDS IDS Sexual and reproductive health and rights Health Key Issues Guide 2006 IDS: Brighton

[B7] Kulczycki A The Abortion Debate in the World Arena 1999 London: Macmillan Press Limited

[B8] Parsons W Public Policy: An Introduction to the Theory and Practice of Policy Analysis 1995 Cheltenham: Edward Elgar Publishing Ltd

[B9] Kingdon J Agendas, Alternatives and Public Policies 2003 2 New York: Longman

[B10] Grindle M Thomas J Public Choices and Policy Change. The Political Economy of Reform in Developing Countries 1991 Baltimore MD: Johns Hopkins University Press

[B11] Sabatier P, Jenkins-Smith H Policy Change and Learning: An Advocacy Coalition Approach. 1993 Boulder: Westview Press

[B12] Keeley J Scoones I Understanding Environmental Policy Processes: Cases from Africa 2003 London: Earthscan

[B13] Roe E Narrative Policy Analysis: Theory and Practice 1994 London and Durham: Duke University Press

[B14] Benhast I Goldstein K Read M The case research strategy in studies in information systems MIS Quarterly 1987 11 3 369 386 10.2307/248684

[B15] Commission African Union Plan of action on sexual and reproductive health rights, 2007-2010 (Maputo Plan of Action) 2006 http://www.unfpa.org/africa/newdocs/maputo_eng.pdf

[B16] Galgalo B Mob attacks gay ‘wedding’ party Daily Nation February 12, 2010, Nairobi http://www.nation.co.ke/News/-/1056/860810/-/vqhvrh/-/index.html

[B17] Galgalo B Murugi urges Kenyans to accept gays Daily Nation October 2, 2010, Nairobi http://www.nation.co.ke/News/Murugi+urges+Kenyans+to+accept+gays+++/-/1056/1024376/-/r8v7oe/-/index.html

[B18] BBC News Malawi gay couple released after presidential pardon BBC News - Africa May 30, 2010 http://www.bbc.co.uk/news/10194057

[B19] Smith A Magga G Uganda’s anti-pornography law to fight homosexual vice Afrik News September 8, 2010 http://www.afrik-news.com/article18213.html

[B20] WHO Unsafe Abortion: Global and Regional Estimates of Incidence of Unsafe Abortion and Associated Mortality in 2003 2007 Geneva: WHO

[B21] Voice of America Kenya debates abortion bill VOA March 12, 2009 http://www.voanews.com/english/news/a-13-2009-03-12-voa28-68679527.html

[B22] Manuh T The passage of domestic violence legislation in Ghana 2007 http://www.dfid.gov.uk/r4d/PDF/Outputs/WomenEmp/GhanaDV.pdf

[B23] Tulloch O Adu-Sarkodie Y Opoku BK Lithur NO Sickle E Delany-Moretlwe S Wambura M Changalucha J Theobald S Using research to influence both policy and practice in the field of sexual and reproductive health: a report from 4 case studies in sub-Saharan Africa Health Research Policy and Systems 2011 9 Suppl 1 S7 10.1186/1478-4505-9-S1-S7 21679377 PMC3121127

[B24] Onyango-Ouma W Ndung’u N Baraza N Birungi H The Making of the Kenya Sexual Offenses Act, 2006: Behind the Scenes 2009 Nairobi: Population Council

[B25] Matathia C Onyango-Ouma W et al The deconstruction of the sexual offences act – 2006 The Making of the Kenya Sexual Offenses Act 2006 Behind the Scenes 2009 Nairobi: Population Council

[B26] AWID Legislating against sexual violence: the Kenyan experience AWID February 12, 2008 http://www.awid.org/Library/Legislating-against-sexual-violence-the-Kenyan-experience2

[B27] Roseman M Reichenbach L Reichenbach L, Roseman MJ Global reproductive health and rights: reflecting on ICPD Reproductive Health and Human Rights: The Way Forward 2009 Philadelphia: University of Pennsylvania Press

[B28] Fischer F Reframing Public Policy: Discursive Politics and Deliberative Practices 2003 Oxford: Oxford University Press

[B29] Thomas L “The politics of the womb”: Kenyan debates over the Affiliation Act Africa Today 2000 47 3/4 150 176

[B30] Crichton J Nyamu-Musembi C John-Langba J Theobald S Towards realising sexual and reproductive health rights in Eastern and Southern Africa: promoting rights and responsibilities beyond the individual Lancet 2006 367 2043 2045 10.1016/S0140-6736(06)68903-4 16798372

[B31] Rein M Schon D Fisher F, Forester J Reframing policy discourse The Argumentative Turn in Policy Analysis and Planning 1993 Durham: Duke University Press 144 166

[B32] Theobald S Tolhurst R Elsey H Standing H Engendering the bureaucracy? Challenges and opportunities for mainstreaming gender in Ministries of Health under sector-wide approaches Health Policy and Planning 2005 20 3 141 149 10.1093/heapol/czi019 15840629

[B33] Crichton J Changing fortunes: analysis of fluctuating policy space for family planning in Kenya Health Policy and Planning 2008 23 339 350 10.1093/heapol/czn020 18653676 PMC2515408

[B34] Izugbara C Undie C Who owns the body? Indigeneous African discourses of the body and contemporary sexual rights rhetoric Reproductive Health Matters 2008 16 31 159 167 10.1016/S0968-8080(08)31344-5 18513617

[B35] Crichton J Nyamu-Musembi C Ngugi A Painful tradeoffs: intimate-partner violence and sexual and reproductive health and rights in Kenya IDS Working Paper 2008 312 Brighton: IDS

[B36] McCombs M Shaw D The agenda-setting function of mass media Public Opinion Quarterly 1972 36 176 187 10.1086/267990

